# Effects of probiotic supplementation on disease progression in patients with moderate to severe chronic kidney disease: a randomized controlled trial

**DOI:** 10.1038/s41598-026-67044-5

**Published:** 2026-09-04

**Authors:** Samar Elshahat Saleh, Sherouk Salah Eldin Elnagar, Mohamed Ashraf Fouda, Nagy Sayed-Ahmed

**Affiliations:** 1 Nephrology and Transplantation Unit, Mansoura Urology and Nephrology Center, Mansoura University, Gomhoria Street, Mansoura, 35516 Egypt; 2 Mansoura Nephrology and Dialysis Unit, Mansoura University, Mansoura, Egypt

**Keywords:** Chronic kidney disease, Probiotics, Lactobacillus plantarum, Estimated glomerular filtration rate, Indoxyl sulfate, Diseases, Medical research, Nephrology

## Abstract

Chronic kidney disease (CKD) affects approximately 10% of the global population, with limited therapeutic options to slow progression in moderate stages. The gut-kidney axis contributes to CKD progression, and probiotics are being investigated as potential therapeutic modulators. This double-blind, randomized, placebo-controlled trial investigated the effect of probiotic supplementation on CKD progression. Seventy-two patients with CKD stages 3 or 4 received either *Lactobacillus plantarum* (10^1^⁰ CFU daily) or placebo for six months. The primary outcome was the change in estimated glomerular filtration rate (eGFR). The probiotic group demonstrated a significant increase in eGFR in contrast to a significant decline in the same variable in the placebo group. Treatment assignment was the sole independent predictor of eGFR change, with benefits observed across CKD stages 3 and 4, and in both diabetic and non-diabetic patients. Urinary indoxyl sulfate levels did not significantly differ between groups, and no correlation was found between its changes and eGFR improvement. No probiotic participants experienced CKD progression, compared to 8.6% in the placebo group. These findings suggest that *Lactobacillus plantarum* supplementation significantly ameliorates eGFR in patients with moderate to severe CKD through mechanisms independent of indoxyl sulfate reduction.

*Trial Registration*: This trial was registered with ClinicalTrials.gov (Registration Number: NCT06863194) Registration date: 13 May 2025.

**Supplementary Information:**

The online version contains supplementary material available at https://doi.org/10.1038/s41598-026-67044-5.

## Introduction

Chronic kidney disease affects approximately 10% of the global population and remains a leading cause of morbidity and mortality worldwide^1^. Progressive decline in renal function is associated with the accumulation of uremic toxins, systemic inflammation, and accelerated cardiovascular disease^2^.

The gut-kidney axis has emerged as a critical mediator of CKD pathophysiology^3,4^. Uremic conditions disrupt intestinal homeostasis, leading to dysbiosis characterized by reduced abundance of beneficial bacteria and expansion of proteolytic species^5^. This altered microbiota promotes bacterial fermentation of aromatic amino acids in the colon, generating protein-bound uremic toxins such as indoxyl sulfate (IS) and p-cresyl sulfate (p-CS)^6,7^. These gut-derived toxins accumulate progressively as renal clearance declines, inducing tubular injury, interstitial fibrosis, oxidative stress, and systemic inflammation processes. Such perturbations directly accelerate kidney function deterioration^8^. Experimental and clinical evidence have demonstrated that elevated IS levels correlate with inflammatory biomarkers and the rate of eGFR decline in CKD patients, establishing uremic toxin reduction as a rational therapeutic target for preserving renal function^9^.

Probiotics are live microorganisms that confer health benefits when administered in adequate amounts. Although preclinical evidence has established mechanistic links between microbiota modulation and reduction in uremic toxins, clinical translation of these findings has been inconsistent. Furthermore, the extent to which microbiota-induced changes in uremic toxin levels directly mediate improvements in glomerular filtration rate remains uncertain. Indeed, existing clinical evidence derives predominantly from patients with advanced renal failure or those already receiving renal replacement therapy^10–12^. In contrast, patients with moderate-stage CKD (stages 3–4) retain substantial residual renal function and represent a potentially optimal target population for disease-modifying interventions; yet this group has remained comparatively underrepresented in probiotic efficacy trials. We conducted a randomized, double-blind, placebo-controlled trial to determine whether six months of *Lactobacillus plantarum* supplementation mitigates the progression of kidney function and whether this is associated with a reduction in urinary indoxyl sulfate in patients with CKD stages 3–4.

## Patients and methods

### Study design and setting

This double-blind, randomized, placebo-controlled trial was conducted at the Urology and Nephrology Center, Mansoura University, Egypt, between May and November 2025. The study protocol was approved by the Medical Research Ethics Committee of Mansoura Faculty of Medicine (approval code: MD.24.06.864, dated July 14, 2024) and prospectively registered at ClinicalTrials.gov (NCT06863194) on 13 May 2025, prior to the enrollment of the first participant. The study was conducted in accordance with the Declaration of Helsinki, and informed consent was obtained from all participants.

No changes were made after trial commencement to the study protocol, trial design, eligibility criteria, interventions, data collection, or prespecified primary and secondary outcomes. Additional post hoc analyses were conducted using the previously collected data. These included baseline-adjusted ANCOVA, parsimonious covariate-adjusted models, an exploratory reconstruction of urinary creatinine excretion, a BMI-change sensitivity analysis, and exploratory sex-stratified analyses. These analyses were not prespecified and are presented as supplementary or exploratory findings.

The full study protocol (approval code: MD.24.06.864) and statistical analysis plan are available upon reasonable request from the corresponding author. A summary of the protocol is also registered at ClinicalTrials.gov (NCT06863194)."

### Participants

Adult patients (≥ 18 years) with CKD stages 3 or 4, defined by eGFR of 15–59 mL/min/1.73 m^2^, were recruited from outpatient nephrology clinics. Exclusion criteria were recent (within 4 weeks) use of probiotics or antibiotics, active malignancy, known structural gastrointestinal disease, or a history of gastrointestinal surgery, pregnancy, and autoimmune disease requiring immunosuppressive therapy.

Sample size was calculated using G*Power software (version 3.1.9.4) based on effect sizes reported by de Araújo et al.^13^. For a two-sample t-test with an alpha error probability of 0.05, a power of 0.80, and an effect size d = 0.67, the required sample size was 72 patients (~ 36 per group).

### Randomization and blinding

Eligible patients were randomly assigned in a 1:1 ratio to receive either probiotic supplementation or a placebo using computer-generated randomization sequences. Allocation concealment was ensured through sequentially numbered, sealed, opaque envelopes maintained by an independent pharmacist. Both participants and all research personnel remained blinded to treatment allocation throughout the study. Probiotic and placebo capsules were identical in appearance to maintain blinding integrity.

### Interventions

**Fig. 1:**
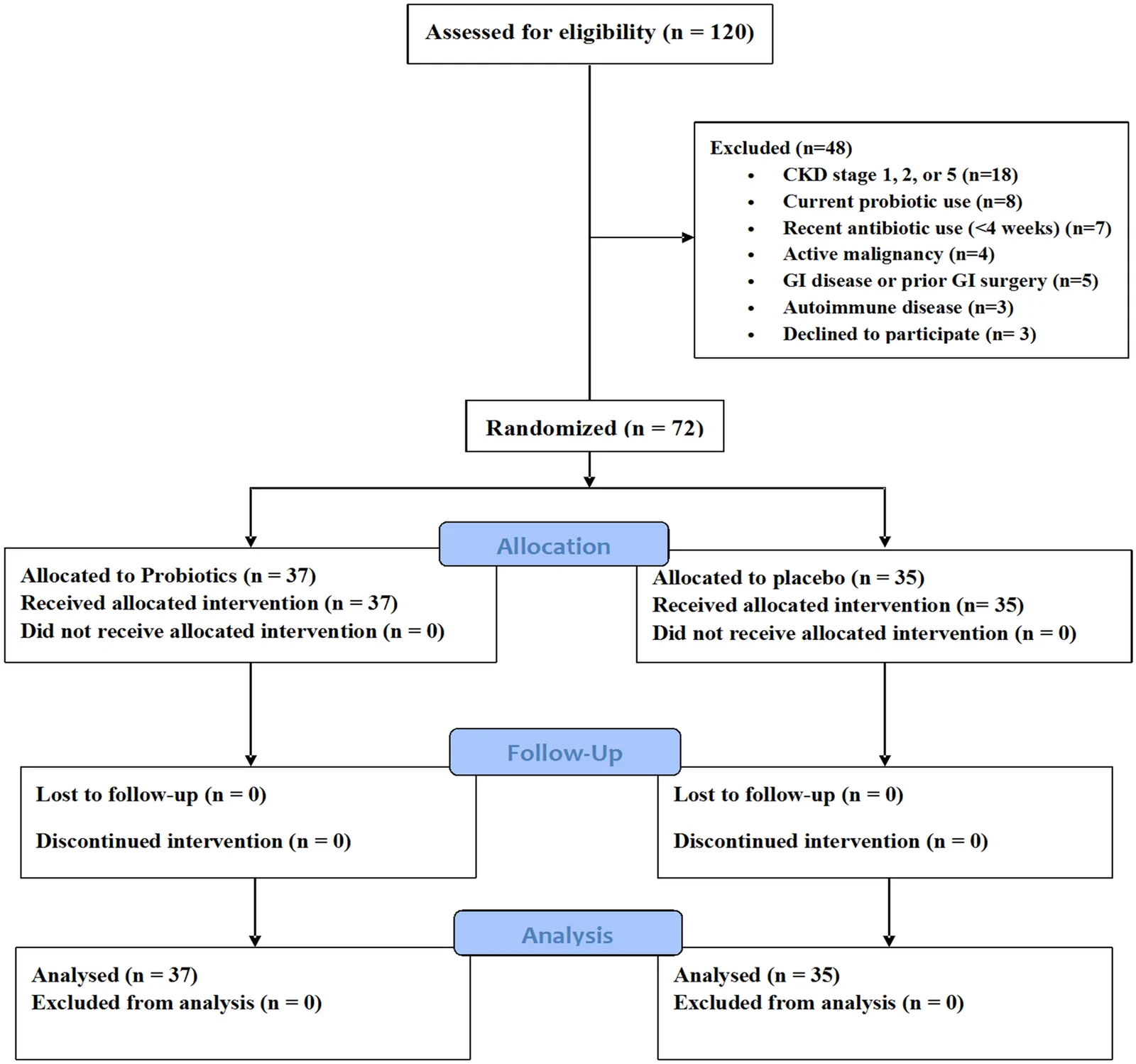
Study flowchart. Flow diagram illustrating participant recruitment, allocation, follow-up, and analysis. Of 120 individuals assessed for eligibility, 48 were excluded based on various criteria, resulting in 72 participants randomized. These participants were allocated to either the Probiotics group (n = 37) or the placebo group (n = 35). All allocated participants received their assigned intervention, completed follow-up, and were included in the final analysis. No participants were lost to follow-up, discontinued the intervention, or were excluded from analysis in either group.

Participants in the intervention group received oral probiotic supplementation containing 10^1^⁰ colony-forming units of *Lactobacillus plantarum* per capsule (Lactogemikan; Pescado Pharmaceuticals, Egypt), administered once daily for 6 months. The control group received matching placebo capsules containing microcrystalline cellulose on the same schedule. Both groups continued standard medical management for CKD, including standardized dietary counseling, as per institutional protocols. Dietary intake was not formally quantified through food frequency questionnaires. All participants with hypertension received a stable angiotensin-converting-enzyme inhibitor or angiotensin-receptor blocker regimen that remained unchanged during follow-up. Treatment adherence was assessed through patient interviews and capsule counts at follow-up visits. The study flowchart is presented in Fig. 1.

### Patient and public involvement

No patients or public were involved in the design, conduct, or reporting of this study.

### Outcome measures

Participants were assessed at baseline and at 6 months, corresponding to the end of the intervention. The primary outcome was the change in eGFR from baseline to 6 months, while secondary outcomes included changes in serum creatinine, 24-h proteinuria, urinary indoxyl sulfate, serum albumin, total cholesterol, and hemoglobin; CKD progression was defined as a ≥ 25% decline in eGFR or doubling of serum creatinine, and additional assessments and analyses included six-month eGFR adjusted for baseline eGFR, 24-h creatinine clearance, reconstructed 24-h urinary creatinine excretion, body mass index (BMI), body weight, blood urea nitrogen (BUN), and treatment-effect estimates according to CKD stage, diabetes status, and sex.

Adverse events were systematically monitored and recorded at each follow-up visit through patient interviews and review of medical records. Harms were defined as any untoward medical occurrence in a participant during the study period, regardless of its causal relationship with the intervention. The severity and relatedness of all reported adverse events to the study intervention were assessed by the study investigators.

### Laboratory procedures

Venous blood samples were collected after an overnight fast at baseline and follow-up visits. Serum creatinine was measured using an enzymatic method with IDMS-traceable calibration on a Roche Cobas automated analyzer. eGFR was calculated using the CKD-EPI 2021 equation. Other biochemical parameters were measured using standard automated methods at the laboratory of Urology and Nephrology Center, Mansoura University, Egypt.

For 24-h urine collection, patients were instructed to discard the first morning void and collect all subsequent urine over the next 24 h. Samples were stored at 4 °C during collection and analyzed within 2 h.

Twenty-four-hour urinary creatinine excretion was reconstructed from the available CrCl and serum creatinine values as urinary creatinine excretion (mg/day) = CrCl (mL/min) × serum creatinine (mg/dL) × 14.4. BUN, BMI, and body weight were recorded at both visits. Twenty-four-hour urinary urea excretion was not measured; BUN was therefore examined as an exploratory serum marker related to urea metabolism and catabolism, but not as a direct quantitative measure of protein intake. Cystatin C was not measured because assay kits were unavailable at our center and the associated procurement costs exceed the available funding for this study.

Urinary indoxyl sulfate was quantified using a commercially available ELISA kit (Human IS ELISA Kit, Catalog ELK9248; detection range 31.25–2000 ng/mL, sensitivity 12.1 ng/mL). First morning midstream urine samples were collected, centrifuged at 2000 × g for 10 min, and either assayed immediately or stored at −20 °C. All samples were analyzed in duplicate.

The ELISA procedure was followed according to the manufacturer's instructions. Briefly, 100 μL of standards (31.25–2000 ng/mL) or samples were added to microtiter wells and incubated at 37 °C for 80 min. After washing three times, 100 μL of biotinylated antibody was added and incubated at 37 °C for 50 min. Following three washes, 100 μL of streptavidin-HRP was added and incubated at 37 °C for 50 min. After five washes, 90 μL of TMB substrate was added, and the mixture was incubated at 37 °C for 20 min. The reaction was stopped with 50 μL of stop solution, and absorbance was measured at 450 nm within 15 min. Standard curves were constructed using power function fitting, with R^2^ > 0.97 required for acceptance. Quality control included duplicate measurements with a coefficient of variation < 15%.

### Statistical analysis

Statistical analyses were performed using SPSS version 26. Normality was assessed using the Shapiro–Wilk test. Normally distributed data is presented as mean ± standard deviation; non-normally distributed data as median with interquartile range (IQR). Categorical variables are expressed as frequencies and percentages.

Baseline characteristics were compared using the Chi-square or Fisher's exact test for categorical variables, and the independent-samples t-test or Mann–Whitney U test for continuous variables. Within-group changes were analyzed using paired t-tests or Wilcoxon signed-rank tests as appropriate. Between-group change-score comparisons were analyzed using independent-samples t tests or Mann–Whitney U tests and were retained as secondary analyses.

For the primary between-group analysis, ANCOVA was performed with eGFR at six months as the dependent variable, treatment group as the main factor, and baseline eGFR as the covariate. Adjusted group means, the adjusted probiotic–placebo difference, 95% confidence interval (CI), *p* value, and partial η^2^ were estimated. Model diagnostics included assessment of residual normality, homoscedasticity, linearity, and multicollinearity.

Separate parsimonious sensitivity models were fitted: baseline eGFR alone; baseline eGFR plus stable ACE inhibitor/angiotensin-receptor blocker use; baseline eGFR plus age and diabetes status; and baseline eGFR plus baseline proteinuria. A further sensitivity model included change in BMI. Sex-stratified treatment estimates and a treatment-by-sex interaction test were added to the subgroup analyses. CKD-stage and diabetes-status interaction tests were also retained. Spearman rank correlation assessed associations between changes in urinary indoxyl sulfate and changes in clinical variables.

All tests were two-tailed, with *p* < 0.05 considered statistically significant. The study was designed to detect the prespecified effect size with 80% power at an alpha level of 0.05.

All 72 randomized participants completed the 6-month intervention period with no dropouts or missing data. The high retention rate was attributed to the comprehensive clinical services and excellent patient care provided by the Urology and Nephrology Center at Mansoura University, which ensured strong participant engagement and adherence to follow-up visits.

## Results

### Baseline characteristics

Seventy-two patients with stages 3 and 4 CKD were randomized to receive either probiotic supplementation (n = 37) or placebo (n = 35) for 6 months. The median age was approximately 60 years, with male predominance (83.8% vs. 80.0%; for probiotic and placebo groups, respectively). Most patients had hypertension (86.5% vs. 94.3%; for probiotic and placebo groups, respectively), and approximately 40% had diabetes mellitus. Baseline median eGFR was 30.8 mL/min/1.73 m^2^ in the probiotic group and 31.7 mL/min/1.73 m^2^ in the placebo group (*p* = 0.45). Stable ACE inhibitor or angiotensin-receptor blocker use was present in 32 of 37 (86.5%) probiotic participants and 33 of 35 (94.3%) placebo participants (*p* = 0.43). Similarly, the other baseline characteristics were not significantly different (all *p* > 0.05) between the two groups (Table 1).

**Table 1:** Baseline characteristics of study participants.

	Probiotic group (n = 37)	Placebo group (n = 35)	*P*-value
Demographics			
Age (years)	57 (49–67)	61 (49.5–65.5)	0.78
Male sex, n (%)	31 (83.8)	28 (80)	0.91
CKD duration (years)	6 (2–8)	6 (3.5–10)	0.41
Anthropometrics			
Weight (kg)	88 (76–105)	92(77–95)	0.99
Body mass index (kg/m^2^)	29.4 (27.2–34.9)	30 (26.2–32.9)	0.80
Comorbidities, n (%)			
Diabetes mellitus	13 (35.1)	16 (45.7)	0.50
Hypertension	32 (86.5)	33 (94.3)	0.43
Stable RAS blockade (ACE inhibitor/ARB), n (%)	32 (86.5)	33 (94.3)	0.43
Current smoking	5 (13.5)	7 (20)	0.68
Baseline renal function			
Serum creatinine (mg/dL)	2.3 (1.9–2.6)	2.2 (1.75–2.55)	0.24
eGFR (mL/min/1.73 m^2^)	30.8 (26.21–37.5)	31.7 (26.23–39.41)	0.45
24-h proteinuria (g/24 h)	0.45 (0.14–0.73)	0.4 (0.24–0.87)	0.75

Data presented as median (interquartile range) for continuous variables and n (%) for categorical variables. CKD = chronic kidney disease; BP = blood pressure; eGFR = estimated glomerular filtration rate. Mann–Whitney U test used for continuous variables; chi-square or Fisher's exact test used for categorical variables. No statistically significant differences were observed between groups (all *p* > 0.05).

### ***Changes in renal function (***Table 2***)***

**Table 2:** Changes in renal function parameters over 6-month intervention.

	Probiotic group (n = 37)	Placebo group (n = 35)	*P*-value†
Serum creatinine (mg/dL)			
Baseline	2.3 (1.9–2.6)	2.2 (1.75–2.55)	0.24
6 months	2.0 (1.7–2.3)	2.3 (1.80–2.70)	0.06
Change from baseline (Δ)	− 0.3 (− 0.4–0.2)	0.1 (0–0.35)	< 0.001
*P*-value*	< 0.001	0.02	–
eGFR (mL/min/1.73 m^2^)			
Baseline	30.8 (26.21–37.5)	31.7 (26.23–39.41)	0.45
6 months	36.1 (31.43–43.64)	29.7 (26.43–35.35)	0.01
Change from baseline (Δ)	4.85 (2.86–7.78)	− 1.5 (− 6.26–0)	< 0.001
*P*-value*	< 0.001	0.009	–
24-h proteinuria (g/24 h)			
Baseline	0.45 (0.14–0.73)	0.40 (0.24–0.87)	0.75
6 months	0.82 (0.57–1.27)	1.09 (0.82–1.34)	0.13
Change from baseline (Δ)	0.42 (0.00–0.55)	0.54 (− 0.03–0.92)	0.19
*P*-value*	0.001	< 0.001	–
CKD progression, n (%)‡	0 (0.0)	3 (8.6)	0.11
Measured 24-h CrCl (mL/min)			
Baseline	41.70 (31.20–55.50)	38.00 (31.25–53.28)	0.77
Six months	45.95 (41.30–56.48)	35.00 (26.50–43.26)	< 0.001
Change	+ 7.80 (2.00–15.95)	− 4.90 (− 10.70 to − 1.05)	< 0.001
Within-group *p* value*	< 0.001	< 0.001	–
Reconstructed 24-h urinary creatinine (mg/day)			
Baseline	1273.7 (1010.9–1918.1)	1185.4 (1004.5–1554.8)	0.34
Six months	1356.5 (1142.3–1766.5)	1117.4 (942.2–1394.7)	0.001
Change	− 0.1 (− 83.1–219.9)	− 50.3 (− 156.4 to − 0.5)	0.012
Within-group *p* value*	0.741	< 0.001	–
BMI (kg/m^2^)			
Baseline	29.4 (27.2–34.9)	30.0 (26.2–32.9)	0.80
Six months	31.0 (27.5–35.0)	31.0 (27.1–33.3)	0.95
Change	0.00 (− 0.62–1.47)	0.39 (− 0.00–1.48)	0.20
Within-group *p* value*	0.316	0.003	–
Body weight (kg)			
Baseline	88.0 (76.0–105.0)	92.0 (77.0–95.0)	0.99
Six months	90.0 (77.5–98.0)	91.76 (79.5–100.5)	0.77
Change	0.0 (− 1.5–4.0)	1.0 (0.0–4.0)	0.16
Within-group *p* value*	0.281	0.004	–
BUN (mg/dL)			
Baseline	27.47 (21.88–35.36)	26.19 (19.45–29.76)	0.14
Six months	23.61 (18.99–28.69)	26.90 (21.29–30.48)	0.10
Change	− 4.72 (− 8.47 to − 2.41)	0.91 (− 1.71–5.64)	< 0.001
Within-group *p* value*	< 0.001	0.232	–

Data presented as median (interquartile range). eGFR = estimated glomerular filtration rate; CKD = chronic kidney disease.

*P-value for within-group comparison (Wilcoxon signed-rank test).

^†^*P*-value for between-group comparison (Mann–Whitney U test).

^‡^Defined as ≥ 25% decline in eGFR or doubling of serum creatinine.

BUN, blood urea nitrogen; CrCl, creatinine clearance; eGFR, estimated glomerular filtration rate.

*Within-group *p* value from the Wilcoxon signed-rank test. †Between-group *p* value from the Mann–Whitney U test; CKD-progression *p* value is from Fisher exact test.

Urinary creatinine excretion was reconstructed as CrCl (mL/min) × serum creatinine (mg/dL) × 14.4

After following up the patients for 6 months, serum creatinine decreased significantly in the probiotic group (median change − 0.3 mg/dL, *p* < 0.001) but increased in the placebo group (median change 0.1 mg/dL, *p* = 0.02), with a significant between-group difference (*p* < 0.001).

Consistent with serum creatinine changes, the probiotic group had a significant increase in eGFR compared to their respective baseline data, with a median increase of 4.85 mL/min/1.73 m^2^ (IQR: 2.86–7.78; *p* < 0.001; Table 2). In contrast, the data of the placebo group showed a median decline in eGFR of − 1.5 mL/min/1.73 m (IQR: − 6.26 to 0; *p* = 0.009) within the same follow-up interval. Furthermore, the difference in the eGFR changes between groups was statistically significant (*p* < 0.001).

On the other hand, both groups experienced significant increases in 24-h proteinuria during the 6-month observation period (0.42 g/24 h, *p* = 0.001; and 0.54 g/24 h, < 0.001; for probiotic and placebo groups, respectively), with no significant difference in the change of proteinuria between the two groups (*p* = 0.19).

Median 24-h CrCl increased from 41.70 to 45.95 mL/min in the probiotic group (median change + 7.80 mL/min; *p* < 0.001) and declined in the placebo group (median change − 4.90 mL/min; *p* < 0.001); the between-group difference in change was significant (*p* < 0.001). Reconstructed 24-h urinary creatinine excretion did not decrease in the probiotic group (1273.7 to 1356.5 mg/day; median change − 0.1 mg/day; *p* = 0.741), whereas it decreased in the placebo group. BUN decreased in the probiotic group (median change − 4.72 mg/dL; *p* < 0.001) and remained statistically stable in the placebo group. In the BMI-change-adjusted ANCOVA, the treatment effect remained significant (*p* < 0.001), whereas BMI change was not a significant predictor (*p* = 0.367). (Table 2).

None of the participants in the probiotic group (compared to three patients (8.6%) in the placebo group) experienced CKD progression, defined as ≥ 25% eGFR decline or creatinine doubling (*p* = 0.11).

### Changes in urinary indoxyl sulfate

Baseline urinary indoxyl sulfate (IS) concentrations were comparable between the probiotic and placebo groups (77.6 vs. 90.65 ng/mL, respectively; *p* = 0.19; Table 3). Albeit non-statistically significant, divergent trajectories in urinary IS levels were observed between the two cohorts; the probiotic group showed a numerical reduction in median urinary IS, whereas the placebo group exhibited a progressive increase, following the six-month intervention. Similarly, the final between-group difference did not reach statistical significance (*p* = 0.15; Table 3).

**Table 3:** Changes in urinary indoxyl sulfate levels (ng/mL) over 6-month intervention.

	Probiotic group (n = 37)	Placebo group (n = 35)	*P*-value†
Urinary indoxyl sulfate (ng/mL)			
Baseline	77.6 (26.1–123)	90.65 (57.2–187.7)	0.19
6 months	57.3 (11.5–146.4)	110 (36.3–149.9)	0.15
Change from baseline (Δ)	0.67 (− 56 to 46.2)	20.7 (− 72.4 to 83.1)	0.77
Percentage change (%)	7.6(− 82.8 to 85.3)	7.5(− 54.3 to 133.1)	0.42
*P*-value*	0.93	0.53	–

Data presented as median (interquartile range).

*P-value for within-group comparison (Wilcoxon signed-rank test).

^†^*P*-value for between-group comparison (Mann–Whitney U test).

### Subgroup analyses

Table 4 presents subgroup analyses examining the consistency of the treatment effect across CKD stages ,whether the patients had concomitant diabetes mellitus (DM) or did not and according to sex. The treatment effect on eGFR change was statistically significant in all CKD subgroups as well as in both diabetic and non-diabetic patients. The increase in eGFR in the probiotic group was statistically significantly higher in stage 3 compared to that in stage 4 CKD patients (*p* = 0.02). Similarly, significant treatment effects were observed in both non-diabetic patients and diabetic patients (*p* < 0.001, *p* < 0.001, respectively), with no significant interaction (*p* = 0.25). In men (n = 59), median eGFR change was + 4.96 mL/min/1.73 m^2^ in the probiotic group and − 2.95 mL/min/1.73 m^2^ in the placebo group (*p* < 0.001). In women (n = 13), the corresponding median changes were + 4.33 and 0.00 mL/min/1.73 m^2^ (*p* = 0.027). Baseline-eGFR-adjusted treatment contrasts were 8.43 mL/min/1.73 m^2^ in men and 4.82 mL/min/1.73 m^2^ in women; the treatment-by-sex interaction was not significant (*p* = 0.193). Because only 13 women were enrolled, the sex-specific estimates are exploratory.

**Table 4:** Subgroup analyses: treatment effect on eGFR change by CKD stage ,DM status and sex.

	Probiotic group	Placebo group	Mean difference in ΔeGFR (95% CI)	*P*-value
According to eGFR				
eGFR 30–59 mL/min/1.73 m^2^ (Stage 3)	6.08 ± 3.63 (n = 21)	−3.77 ± 5.09 (n = 22)	9.85 (95% CI 7.13, 12.57)	< 0.001
eGFR 15–29 mL/min/1.73 m^2^ (Stage 4)	4.74 ± 3.18 (n = 16)	−0.22 ± 5.17 (n = 13)	4.96 (95% CI 1.51, 8.41)	0.007
P for interaction	–	–	–	0.02
According to DM Status				
Non-diabetic patients	5.13 ± 3.14 (n = 24)	−4.00 ± 5.92 (n = 19)	9.13 (95% CI 6.04, 12.22)	< 0.001
Diabetic patients	6.06 ± 4.01 (n = 13)	−0.61 ± 3.98 (n = 16)	6.67 (95% CI 3.60, 9.74)	< 0.001
P for interaction	–	–	–	0.25
According to Sex				
Men	+ 4.96 (2.79–8.22); n = 31	− 2.95 (− 7.04–0.00); n = 28	8.43 (5.99–10.86), adjusted	< 0.001
Women	+ 4.33 (3.38–5.16); n = 6	0.00 (− 1.74–1.27); n = 7	4.82 (0.65–8.99), adjusted	0.027
P for interaction	–	–	–	0.193

Data presented as mean ± SD. eGFR = estimated glomerular filtration rate (mL/min/1.73 m^2^); CKD = chronic kidney disease; CI = confidence interval. The mean difference represents the difference between the probiotic and placebo groups. An independent-samples t-test was used to compare groups within each subgroup. Interaction was tested using linear regression with the treatment × subgroup interaction term.

In the baseline-adjusted ANCOVA, adjusted mean eGFR at six months was 38.90 mL/min/1.73 m^2^ in the probiotic group and 31.18 mL/min/1.73 m^2^ in the placebo group, corresponding to an adjusted difference of 7.72 mL/min/1.73 m^2^ (95% CI 5.63–9.82; *p* < 0.001; partial η^2^ = 0.439; Table 5). The treatment estimate remained significant in the separate parsimonious models that additionally included stable RAS blockade, age plus diabetes, baseline proteinuria, or BMI change (all *p* < 0.001).

**Table 5:** Parsimonious ANCOVA models for treatment effect on eGFR at six months.

Parsimonious ANCOVA model	Treatment estimate (mL/min/1.73 m2)	95% CI	*p* value
Model 1: Baseline eGFR	7.72	5.63–9.82	< 0.001
Model 2: Baseline eGFR + stable RAS blockade	7.83	5.47–10.19	< 0.001
Model 3: Baseline eGFR + age + diabetes	7.99	5.91–10.06	< 0.001
Model 4: Baseline eGFR + baseline proteinuria	7.71	5.58–9.83	< 0.001
Model 5: Baseline eGFR + BMI change	7.56	5.43–9.69	< 0.001

The dependent variable was six-month eGFR. Each model included treatment group and baseline eGFR; additional covariates are specified by row. The treatment estimate is the adjusted probiotic–placebo difference in mL/min/1.73 m^2^. CI, confidence interval; DM, diabetes mellitus; RAS, renin–angiotensin system.CI = confidence interval; eGFR = estimated glomerular filtration rate.

All variance inflation factor values are < 5, indicating no multicollinearity concerns.

To assess the relationship between changes in urinary indoxyl sulfate (IS) and kidney function and other laboratory data, a correlation analysis was conducted. No significant correlation was observed between changes in indoxyl sulfate and changes in kidney function or any of the other laboratory data studied. Specifically, the changes in eGFR failed to show significant correlation with changes in indoxyl sulfate (ρ = 0.02, *p* = 0.86), indicating that indoxyl sulfate reduction might not be a significant mediator of the observed improvement in renal function.

### Safety and tolerability

During the 6-month intervention period, no adverse events, side effects, or unintended harms were reported or observed in either the probiotic or placebo group. All participants tolerated the assigned interventions well, and no participants discontinued the study due to safety concerns.

This randomized trial found that six months of L. plantarum supplementation was associated with higher creatinine-based eGFR at follow-up than placebo. The baseline-adjusted ANCOVA and the directionally concordant 24-h creatinine-clearance analysis support the primary finding, while the reconstructed urinary creatinine, BMI, and weight findings do not suggest a loss of body mass or creatinine production in the probiotic group. On the other hand, urinary indoxyl sulfate levels remained unchanged in both groups, with no correlation between changes in indoxyl sulfate and improvements in eGFR.

## Discussion

The magnitude of eGFR improvement observed in our trial is particularly noteworthy when contextualized within the existing literature, which presents a complex landscape of both supporting and contradicting evidence. Our findings align with recent meta-analyses demonstrating that probiotics significantly reduce serum creatinine and blood urea nitrogen and effectively decrease the rate of renal function decline^14^. These positive outcomes are further confirmed by recent systematic reviews emphasizing the role of multi-strain probiotic formulations in optimizing CKD management and improving metabolic profiles^15,16^.

However, our results diverge from other studies that have reported non-significant effects of biotic supplements on renal function. A 2022 meta-analysis of 23 RCTs found that while biotics improved antioxidative capacity and reduced IL-6, they did not significantly modify eGFR or serum albumin^11^. A 2023 Cochrane review concluded that the evidence for probiotics in preserving kidney function remains of very low certainty^17^. Most recently, a 2025 clinical study of 23 patients with advanced stage 5 CKD observed no significant changes in serum creatinine or eGFR after six months of probiotic supplementation^18^.

These discrepancies may be attributed to several critical factors, including strain specificity, intervention duration, and the targeted CKD stage. The specific *Lactobacillus plantarum* strain used in our study has demonstrated unique renoprotective properties in experimental models and significantly attenuated renal injury and fibrotic-related proteins^19–21^. In contrast, many meta-analyses utilized diverse or multispecies formulations with potentially lower metabolic potency. Furthermore, our six-month intervention duration was longer than many previous trials, potentially allowing sufficient time for physiological changes in eGFR to manifest. Most importantly, our study focused on patients with moderate-to-severe CKD (stages 3 or 4), whereas studies reporting no benefit often involved patients with advanced stage 5 CKD or those already on dialysis^18^. At these later stages, irreversible structural damage and profound metabolic derangements may limit the efficacy of gut-targeted interventions. Our findings suggest that earlier intervention before the onset of end-stage renal disease may yield superior clinical outcomes.

Indoxyl sulfate is a prototypical gut-derived, protein-bound uremic toxin that originates from the bacterial metabolism of dietary tryptophan in the colon. Under physiological conditions, IS is efficiently cleared by the kidneys through active tubular secretion^22^. As renal function declines, urinary excretion of IS decreases, and it progressively accumulates in the systemic circulation, reaching concentrations that could exert potent nephrotoxic effects^23^. In this context, recent evidence has highlighted that IS is not merely a marker of kidney dysfunction but a primary driver of disease progression, as it induces oxidative stress and pro-inflammatory signaling within renal proximal tubular cells^24^, leading to the activation of the renin–angiotensin–aldosterone system and the induction of epithelial-to-mesenchymal transition^25^.These molecular pathways culminate in accelerated interstitial fibrosis and glomerulosclerosis, creating a vicious cycle where toxin accumulation further impairs the residual nephron population^8^.

Indoxyl sulfate has been a target for therapeutic trials aiming at delaying the progression of renal disease and ameliorating the burden of uremic toxicity. Among the options used to mitigate IS production, decreasing dietary protein as well as the Mediterranean diet have been the main therapeutic advice. While probiotics have been recently introduced to control IS^26^. However, the therapeutic potential of probiotics to reduce IS levels remains a subject of significant debate in current literature. Recent publications in 2024 and 2025 have reported divergent outcomes; some demonstrated a significant reduction in serum IS, while others, including large-scale systematic reviews, found that probiotic effects on protein-bound toxins are often marginal and strain-specific^12,27^.

A central and intriguing finding of our study is that the observed renal benefit occurred independently of changes in urinary indoxyl sulfate levels. Although the probiotic group showed a numerical reduction in urinary IS compared to the progressive increase in the placebo group, the difference between groups did not reach statistical significance, and no correlation was found between urinary IS changes and eGFR improvement. This lack of concordance is consistent with the SYNERGY trial and other recent studies, which reported that while synbiotics ameliorated CKD progression, their impact on IS was negligible^14,28^. Furthermore, a systematic review published in 2021 has reinforced the absence of an intimate relationship between IS reduction and improvement of kidney function following probiotic therapy^29^. Collectively, these findings suggest that the renoprotective effects of *Lactobacillus plantarum* could be mediated by alternative pathways beyond the simple reduction of gut-derived uremic toxins.

The absence of a significant urinary IS decrease in the probiotic group reflects a complex interplay between toxin production and renal clearance^30^. It would be speculated that improved eGFR enhances renal capacity for toxin elimination^31^. Even if probiotic intervention reduces gut IS production, the concomitant increase in glomerular filtration and tubular secretion is expected to maintain or increase the absolute amount of IS excreted, masking the decrease in urinary concentration^28^. Conversely, declining renal function that has been observed in the placebo group would lead to elevated serum IS levels^22^. Despite the reduced glomerular filtration capacity, this elevated systemic toxin burden would paradoxically increase urinary excretion as the remaining nephrons attempt to clear the mounting toxic load^32^; denoting that the urinary IS can be a reflection of the balance between its systemic burden and the renal excretory efficiency, rather than gut production alone.

Another mechanism by which probiotics can ameliorate the progression of CKD in discordance with its effect on IS may involve enhancement of intestinal barrier integrity and reduction of systemic inflammation that are commonly encountered in advanced CKD patients. CKD is characterized by a leaky gut phenotype, where disruption of tight junction proteins allows translocation of endotoxins and microbial products into systemic circulation, triggering inflammatory responses^3,4,6^. Probiotics can upregulate tight junction proteins and restore the intestinal barrier, interrupting this cycle of inflammation and renal injury^33,34^. Additionally, it has been shown that short-chain fatty acids (SCFAs) produced by beneficial microbes may protect the glomerular barrier and attenuate renal fibrosis through G protein-coupled receptor activation^3,35^. While inflammatory biomarkers and SCFA levels were not measured in this study, these pathways represent plausible mechanisms for the observed eGFR improvement and warrant further investigation.

Several biologically plausible pathways merit future study *Lactobacillus plantarum* may modulate systemic inflammation, strengthen intestinal-barrier integrity and reduce endotoxemia, alter short-chain-fatty-acid production in ways that influence renal hemodynamics, or reduce other unmeasured uremic toxins^3,33–35^These mechanisms were not directly evaluated in this trial and remain hypothesis-generating.

The measured 24-h creatinine-clearance result was directionally consistent with the eGFR result, while reconstructed urinary creatinine excretion, BMI, and body weight did not decrease in the probiotic group. Together, these findings argue against a simple explanation based solely on reduced creatinine production. Nevertheless, creatinine clearance and reconstructed urinary creatinine remain creatinine-based measures and are affected by collection quality and renal tubular secretion.

The observed increase in 24-h proteinuria in both groups, despite eGFR improvement in the probiotic cohort, requires careful interpretation. Since this increase was independent of treatment allocation, it likely reflects the natural progression of glomerular barrier damage or characteristic variability in 24-h urine collection protocols. Some researchers have suggested that an improved filtration rate might paradoxically increase the passage of proteins across a damaged glomerular basement membrane due to altered intra-glomerular hemodynamics^36,37^.

The current study elegantly adopted substantial methodological rigor through a double-blind, randomized, placebo-controlled design with 100% retention; thus, eliminating selection and attrition bias. The consistency of treatment effects across CKD stages and diabetes mellitus status denotes generalizability of the findings, and use of baseline-adjusted ANCOVA with several parsimonious sensitivity models. Enrollment of moderate-to-severe CKD patients (stages 3 or 4) who have preserved residual kidney function with a possible degree of potential reversibility, combined with a six-month intervention duration, strengthens our findings.

However, several limitations warrant acknowledgment. The single-center design limits external validity. Inflammatory markers and gut microbiome composition were not assessed, precluding direct evaluation of the proposed alternative mechanisms. Indoxyl sulfate was quantified in urine by ELISA rather than in serum^28,38^, as urinary excretion may not accurately reflect the systemic toxin burden. Cystatin C and exogenous-marker-measured GFR were unavailable.

In conclusion, *Lactobacillus plantarum* supplementation at 10^1^⁰ CFU daily for six months may significantly improve eGFR in patients with CKD stages 3 and 4, which is consistent across CKD stages and DM status, suggesting a renal protective role that might be independent of uremic toxin reduction.

## Practical application

Daily supplementation with *Lactobacillus plantarum* (10^1^⁰ CFU) for six months may serve as a safe and effective adjuvant therapy to slow CKD progression in patients with stages 3 or 4. The results of the current study may support incorporating specific probiotic strains into the nutritional management of non-dialysis CKD patients. Probiotic therapy can be considered a part of a comprehensive metabolic and nutritional strategy, particularly in stage 3 patients, where the benefit is most pronounced.

## Supplementary Information

Supplementary Information 1.

Supplementary Information 2.

Supplementary Information 3.

## Data Availability

The datasets generated or analyzed during the current study are not publicly available due to their confidential nature and institutional policies. However, they are available from the corresponding author on reasonable request, subject to institutional and governance approval for data sharing.

## Declarations

### Use of AI and AI-assisted technologies

During the preparation of this manuscript, the authors used ChatGPT (OpenAI) to assist with language editing and refinement. The tool was not used to design the study, analyze data, interpret results, or draw conclusions. All content was reviewed and verified by the authors, who take full responsibility for the final manuscript.
