# Supplementary material for: Effects of probiotic supplementation on disease progression in patients with moderate to severe chronic kidney disease: a randomized controlled trial
Source: Sci Rep. 2026 Sep 4;16:27799. doi: 10.1038/s41598-026-67044-5 (PMC13545210; doi:10.1038/s41598-026-67044-5)
Supplement: Supplementary file 1 [file 41598_2026_67044_MOESM3_ESM.pdf]

Supplementary Table 1: Spearman Correlation Between Change in Urinary Indoxyl Sulfate and Changes in Clinical Parameters[1]

**Parameter**	**Spearman ρ[2]**	**P-value**
Change in eGFR (mL/min/1.73 m^2)	0.020	0.86
Change in serum creatinine (mg/dL)	-0.055	0.65
Change in proteinuria (g/24h)	0.052	0.66
Change in serum albumin (g/dL)	-0.085	0.48
Change in total cholesterol (mg/dL)	-0.185	0.12
Change in hemoglobin (g/dL)	0.059	0.62
Change in ferritin (ng/mL)	0.201	0.09

[1]Δ denotes change from baseline to 6 months. Negative correlation indicates an inverse relationship.

[2]Spearman's rank correlation coefficient was used due to the non-normal distribution of variables. N = 72 patients.
