# Supplementary material for: Effects of probiotic supplementation on disease progression in patients with moderate to severe chronic kidney disease: a randomized controlled trial
Source: Sci Rep. 2026 Sep 4;16:27799. doi: 10.1038/s41598-026-67044-5 (PMC13545210; doi:10.1038/s41598-026-67044-5)
Supplement: Supplementary file 3 [file 41598_2026_67044_MOESM2_ESM.docx]

RESEARCH PROTOCOL

Effects of Probiotic Supplementation on Disease Progression in Patients with Moderate to Severe Chronic Kidney Disease: A Randomized Controlled Trial

Protocol Title: Effects of Probiotic Supplementation on Disease Progression in Patients with Moderate to Severe Chronic Kidney Disease: A Randomized Controlled Trial

Protocol Identifying Number: MD.24.06.864

Date of Protocol: July 14, 2024

Date of Last Amendment: N/A (Original protocol, no amendments)

PART 1: RESEARCH PROTOCOL

1. PROJECT SUMMARY

This double-blind, randomized, placebo-controlled trial investigates the effect of probiotic supplementation on disease progression in patients with moderate to severe chronic kidney disease (CKD stages 3-4). The gut-kidney axis has emerged as a critical mediator of CKD pathophysiology, with dysbiosis contributing to the production of uremic toxins such as indoxyl sulfate (IS). Although preclinical evidence supports microbiota modulation as a therapeutic target, clinical translation has been inconsistent, particularly in patients with moderate-stage CKD who retain substantial residual renal function. This study will enroll 72 patients (36 per group) with CKD stages 3 or 4 (eGFR 15-59 mL/min/1.73 m²) to receive either Lactobacillus plantarum (10¹⁰ CFU daily) or placebo for six months. The primary outcome is the change in estimated glomerular filtration rate (eGFR) from baseline to 6 months. Secondary outcomes include changes in serum creatinine, 24-hour proteinuria, urinary indoxyl sulfate, serum albumin, total cholesterol, and hemoglobin. The study will evaluate whether probiotic supplementation ameliorates kidney function decline and whether this effect is mediated by reduction in uremic toxins. Results from this trial will provide evidence for a potential disease-modifying intervention in moderate-stage CKD and clarify the mechanisms underlying microbiota-mediated renal protection.

Duration of Study: 6 months of intervention + 1 month for recruitment and baseline assessment = approximately 7 months total

Expected Outcomes: Demonstration that probiotic supplementation significantly improves eGFR in patients with moderate to severe CKD, with potential mechanisms independent of uremic toxin reduction.

2. GENERAL INFORMATION

2.1 Protocol Title, Number, and Date

- Title: Effects of Probiotic Supplementation on Disease Progression in Patients with Moderate to Severe Chronic Kidney Disease: A Randomized Controlled Trial
- Protocol Number: MD.24.06.864
- Date Approved by Ethics Committee: July 14, 2024

2.2 Sponsor/Funder

- Sponsor: Urology and Nephrology Center, Mansoura University, Egypt
- Address: Gomhoria Street, Mansoura, 35516, Egypt
- Funding: This study is funded as part of an MD research program by the Urology and Nephrology Center, Mansoura University, Egypt. No external commercial or industry funding is received.

2.3 Investigator(s) and Research Site(s)

| Role | Name | Title | Address | Telephone | Email | Responsibilities |
| --- | --- | --- | --- | --- | --- | --- |
| Principal Investigator | Dr. Samar Elshahat Saleh | MSc, MBBCh | Nephrology and Transplantation Unit, Mansoura Urology and Nephrology Center, Egypt | +201023771331 | [samarelshahat@mans.edu.eg](mailto:samarelshahat@mans.edu.eg) | Study design, participant recruitment, data collection, manuscript preparation |
| Co-Investigator | Dr. Nagy Sayed-Ahmed | Professor of Internal Medicine & Nephrology | Mansoura Nephrology and Dialysis Unit, Mansoura University, Egypt | 01550140078 | nagysayedahmed@mans.edu.eg | Study supervision, critical review of the manuscript |
| Co-Investigator | Dr. Sherouk Salah Eldin Elnagar | Lecturer of Internal Medicine & Nephrology | Mansoura Nephrology and Dialysis Unit, Mansoura University, Egypt | 01093334767 | Sherouk43@gmail.com | Data analysis and interpretation |
| Co-Investigator | Dr. Mohamed Ashraf Fouda | Consultant of Internal Medicine & Nephrology | Nephrology and Transplantation Unit, Mansoura Urology and Nephrology Center, Egypt | 01114050409 | Afouda7358@yahoo.com | Data analysis and interpretation |

Research Site: Urology and Nephrology Center, Mansoura University, Egypt

2.4 Clinical Laboratory and Other Institutions

- Laboratory: Laboratory of Urology and Nephrology Center, Mansoura University, Egypt
  - Address: Gomhoria Street, Mansoura, 35516, Egypt
  - Responsibilities: Serum creatinine measurement, eGFR calculation, biochemical parameter analysis, urinary indoxyl sulfate quantification
- Ethics Committee: Medical Research Ethics Committee of Mansoura Faculty of Medicine
  - Approval Code: MD.24.06.864
  - Date of Approval: July 14, 2024

3. RATIONALE AND BACKGROUND INFORMATION

3.1 Problem Statement

Chronic kidney disease (CKD) affects approximately 10% of the global population and remains a leading cause of morbidity and mortality worldwide. Progressive decline in renal function is associated with the accumulation of uremic toxins, systemic inflammation, and accelerated cardiovascular disease. The prognosis for patients with moderate-stage CKD (stages 3-4) is particularly concerning, as many progress to end-stage renal disease requiring dialysis or transplantation. Current therapeutic options to slow CKD progression are limited, and novel disease-modifying interventions are urgently needed.

3.2 Current Knowledge and Relevant Studies

Recent evidence has identified the gut-kidney axis as a critical mediator of CKD pathophysiology. Uremic conditions disrupt intestinal homeostasis, leading to dysbiosis characterized by reduced abundance of beneficial bacteria and expansion of proteolytic species. This altered microbiota promotes bacterial fermentation of aromatic amino acids in the colon, generating protein-bound uremic toxins such as indoxyl sulfate (IS) and p-cresyl sulfate (p-CS). These gut-derived toxins accumulate progressively as renal clearance declines, inducing tubular injury, interstitial fibrosis, oxidative stress, and systemic inflammation processes that directly accelerate kidney function deterioration.

Experimental and clinical evidence have demonstrated that elevated IS levels correlate with inflammatory biomarkers and the rate of eGFR decline in CKD patients, establishing uremic toxin reduction as a rational therapeutic target for preserving renal function. Probiotics are live microorganisms that confer health benefits when administered in adequate amounts. Although preclinical evidence has established mechanistic links between microbiota modulation and reduction in uremic toxins, clinical translation of these findings has been inconsistent.

3.3 Rationale for This Study

The extent to which microbiota-induced changes in uremic toxin levels directly mediate improvements in glomerular filtration rate remains uncertain. Existing clinical evidence derives predominantly from patients with advanced renal failure or those already receiving renal replacement therapy. In contrast, patients with moderate-stage CKD (stages 3-4) retain substantial residual renal function and represent a potentially optimal target population for disease-modifying interventions; yet this group has remained comparatively underrepresented in probiotic efficacy trials.

This study will address this gap by investigating whether probiotic supplementation mitigates the progression of kidney function in patients with moderate to severe CKD and whether this is associated with a reduction in urinary indoxyl sulfate.

4. STUDY GOALS AND OBJECTIVES

4.1 Primary Objective To determine whether six months of probiotic supplementation significantly improves estimated glomerular filtration rate (eGFR) compared to placebo in patients with CKD stages 3-4.

4.2 Secondary Objectives

1 To evaluate the effect of Lactobacillus plantarum supplementation on serum creatinine levels
2 To assess changes in 24-hour proteinuria
3 To measure changes in urinary indoxyl sulfate levels
4 To evaluate changes in serum albumin, total cholesterol, and hemoglobin
5 To determine the incidence of CKD progression (defined as ≥25% decline in eGFR or doubling of serum creatinine)
6 To assess the consistency of treatment effects across CKD stages (3 vs. 4) and diabetes status
7 To identify independent predictors of eGFR improvement
8 To evaluate the relationship between changes in urinary indoxyl sulfate and clinical parameters

5. STUDY DESIGN

5.1 Type of Study Double-blind, randomized, placebo-controlled trial

5.2 Research Population Adult patients (≥18 years) with CKD stages 3 or 4, defined by eGFR of 15-59 mL/min/1.73 m², recruited from outpatient nephrology clinics at the Urology and Nephrology Center, Mansoura University, Egypt.

5.3 Inclusion Criteria

- Age ≥18 years
- Confirmed CKD stage 3 or 4 (eGFR 15-59 mL/min/1.73 m²)
- Stable renal function (no acute kidney injury in the preceding 3 months)
- Willing and able to provide informed consent

5.4 Exclusion Criteria

- Recent (within 4 weeks) use of probiotics or antibiotics
- Active malignancy
- Known structural gastrointestinal disease
- History of gastrointestinal surgery
- Pregnancy or lactation
- Autoimmune disease requiring immunosuppressive therapy
- Inability to comply with study procedures

5.5 Withdrawal Criteria Participants may be withdrawn from the study if they:

- Develop serious adverse events related to study intervention
- Become pregnant during the study
- Develop acute kidney injury
- Require emergency dialysis or renal transplantation
- Withdraw informed consent
- Are unable to comply with study procedures

5.6 Sample Size Sample size was calculated using G*Power software (version 3.1.9.4) based on effect sizes reported by de Araújo et al. For a two-sample t-test with an alpha error probability of 0.05, a power of 0.80, and an effect size d = 0.67, the required sample size was 72 patients (~36 per group)

5.7 Expected Duration of Study

- Recruitment and baseline assessment: 1 month
- Intervention period: 6 months
- Total study duration: Approximately 7 months

6. STUDY DESIGN AND PROCEDURES

6.1 Study Flow Diagram

6.2 Randomization Eligible patients will be randomly assigned in a 1:1 ratio to receive either probiotic supplementation or placebo using computer-generated randomization sequences. Allocation concealment will be ensured through sequentially numbered, sealed, opaque envelopes maintained by an independent pharmacist. This method ensures that neither the participants nor the study personnel can predict or influence treatment assignment.

6.3 Blinding Both participants and all research personnel will remain blinded to treatment allocation throughout the study. Probiotic and placebo capsules will be identical in appearance to maintain blinding integrity. Only the independent pharmacist will have access to the randomization code, which will be kept in a sealed envelope and only opened in case of medical emergency or upon study completion.

6.4 Intervention Period The intervention period will last 6 months, during which participants will receive their assigned treatment (probiotic or placebo) once daily.

7. METHODOLOGY

7.1 Interventions

Probiotic Group:

- Intervention: Oral probiotic supplementation containing 10¹⁰ colony-forming units (CFU) of Lactobacillus plantarum per capsule
- Product Name: Lactogemikan
- Manufacturer: Pescado Pharmaceuticals, Egypt
- Dosage: One capsule daily
- Duration: 6 months
- Administration: Participants will be instructed to take the capsule with water, preferably in the morning with breakfast.

Placebo Group:

- Intervention: Matching placebo capsules containing microcrystalline cellulose
- Dosage: One capsule daily
- Duration: 6 months
- Administration: Identical to probiotic group

Concomitant Medications: Both groups will continue standard medical management for CKD, including:

- Antihypertensive medications as prescribed
- Statins as indicated
- Phosphate binders if needed
- Standardized dietary counseling as per institutional protocols

7.2 Procedures

Baseline Assessment (Week 0):

- Demographic data collection (age, sex, occupation)
- Medical history (comorbidities, medications, previous surgeries)
- Physical examination (blood pressure, heart rate, body weight, height)
- Laboratory investigations (see Section 7.3)
- Informed consent verification
- Randomization and treatment allocation
- Dispensing of study medication with instructions

Follow-up Visits:

- Week 2: Telephone contact to assess tolerability and adherence
- Month 3: In-person visit for safety assessment and adherence evaluation
- Month 6: Final assessment with repeat laboratory investigations

End-of-Study Assessment (Month 6):

- Repeat physical examination
- Repeat laboratory investigations (see Section 7.3)
- Adverse event assessment
- Medication adherence evaluation
- Study medication collection and count
- Final questionnaire

7.3 Laboratory Procedures

Venous Blood Samples:

- Collected after an overnight fast at baseline and 6-month follow-up
- Serum creatinine measured using an enzymatic method with IDMS-traceable calibration on a Roche Cobas automated analyzer
- eGFR calculated using the CKD-EPI 2021 equation
- Other biochemical parameters (serum albumin, total cholesterol, hemoglobin) measured using standard automated methods at the laboratory of Urology and Nephrology Center, Mansoura University, Egypt

24-Hour Urine Collection:

- Patients instructed to discard the first morning void and collect all subsequent urine over the next 24 hours
- Samples stored at 4°C during collection and analyzed within 2 hours
- Proteinuria measured using standard laboratory methods

Urinary Indoxyl Sulfate Measurement:

- First morning midstream urine samples collected
- Samples centrifuged at 2000×g for 10 minutes
- Either assayed immediately or stored at -20°C
- Urinary indoxyl sulfate quantified using a commercially available ELISA kit (Human IS ELISA Kit, Catalog ELK9248; detection range 31.25-2000 ng/mL, sensitivity 12.1 ng/mL)
- All samples analyzed in duplicate
- ELISA procedure followed according to manufacturer's instructions:
  - 100 μL of standards (31.25-2000 ng/mL) or samples added to microtiter wells
  - Incubated at 37°C for 80 minutes
  - After washing three times, 100 μL of biotinylated antibody added and incubated at 37°C for 50 minutes
  - Following three washes, 100 μL of streptavidin-HRP added and incubated at 37°C for 50 minutes
  - After five washes, 90 μL of TMB substrate added and incubated at 37°C for 20 minutes
  - Reaction stopped with 50 μL of stop solution
  - Absorbance measured at 450 nm within 15 minutes
- Standard curves constructed using power function fitting with R² >0.97 required for acceptance
- Quality control included duplicate measurements with a coefficient of variation <15%

7.4 Treatment Adherence Assessment Treatment adherence will be assessed through:

- Patient interviews at each visit
- Capsule counts at follow-up visits (participants will return all capsules, including empty blister packs)
- Participant diary (optional, to record daily medication intake)
- Calculation of adherence percentage: (number of capsules taken / number of capsules prescribed) × 100

8. OUTCOME MEASURES

8.1 Primary Outcome

- Change in eGFR from baseline to 6 months: Calculated as eGFR at 6 months minus eGFR at baseline (mL/min/1.73 m²)

8.2 Secondary Outcomes

- Change in serum creatinine (mg/dL)
- Change in 24-hour proteinuria (g/24 h)
- Change in urinary indoxyl sulfate (ng/mL)
- Change in serum albumin (g/dL)
- Change in total cholesterol (mg/dL)
- Change in hemoglobin (g/dL)
- Incidence of CKD progression, defined as ≥25% decline in eGFR or doubling of serum creatinine

8.3 Safety Outcomes

- Incidence and severity of adverse events
- Incidence and severity of serious adverse events
- Incidence of treatment discontinuation due to adverse events

9. ADVERSE EVENTS MONITORING

9.1 Definition of Adverse Events An adverse event (AE) is defined as any untoward medical occurrence in a participant during the study period, regardless of its causal relationship with the study intervention.

9.2 Definition of Serious Adverse Events A serious adverse event (SAE) is defined as any adverse event that:

- Results in death
- Is life-threatening
- Results in hospitalization or prolongation of existing hospitalization
- Results in persistent or significant disability/incapacity
- Consists of a congenital anomaly or birth defect
- Is otherwise considered serious by the investigator

9.3 Monitoring and Recording

- Adverse events will be systematically monitored and recorded at each follow-up visit through patient interviews and review of medical records
- The severity and relatedness of all reported adverse events to the study intervention will be assessed by the study investigators
- All serious adverse events will be reported to the ethics committee within 24 hours of occurrence

9.4 Stopping Rules The study will be stopped for an individual participant if:

- A serious adverse event related to the study intervention occurs
- The participant experiences acute kidney injury
- The participant requires emergency dialysis or renal transplantation
- The participant withdraws informed consent

10. DATA MANAGEMENT AND STATISTICAL ANALYSIS

10.1 Data Management

- Data will be collected on standardized case report forms (CRFs)
- All data will be entered into a secure, password-protected database
- Data quality will be ensured through regular monitoring and verification
- Missing data will be documented and handled according to pre-specified rules
- Data will be analyzed using SPSS version 26

10.2 Statistical Methods

Descriptive Statistics:

- Normality will be assessed using the Shapiro-Wilk test
- Normally distributed data will be presented as mean ± standard deviation
- Non-normally distributed data will be presented as median with interquartile range (IQR)
- Categorical variables will be expressed as frequencies and percentages

Baseline Comparisons:

- Baseline characteristics will be compared between groups using:
  - Chi-square or Fisher's exact test for categorical variables
  - Independent-samples t-test or Mann-Whitney U test for continuous variables

Within-Group Changes:

- Within-group changes from baseline to 6 months will be analyzed using:
  - Paired t-tests for normally distributed data
  - Wilcoxon signed-rank tests for non-normally distributed data

Between-Group Comparisons:

- Between-group differences in change from baseline will be assessed using:
  - Independent samples t-tests for normally distributed data
  - Mann-Whitney U tests for non-normally distributed data

Subgroup Analyses:

- Treatment effects will be evaluated across CKD stage (3 vs. 4) and diabetes status (present vs. absent)
- Interactions between treatment and subgroup variables will be tested using linear regression models with interaction terms

Multivariate Analysis:

- Multivariate linear regression analysis will be used to identify independent predictors of eGFR change
- Model will adjust for age, baseline eGFR, diabetes mellitus, and baseline proteinuria
- Model diagnostics will include assessment of multicollinearity using variance inflation factors (VIF < 5 considered acceptable)

Correlation Analysis:

- Spearman's rank correlation coefficient will be used to assess correlations between changes in urinary indoxyl sulfate and clinical parameters

Significance Level:

- All tests will be two-tailed with p<0.05 considered statistically significant

10.3 Sample Size Justification The study was designed to detect the prespecified effect size with 80% power at an alpha level of 0.05. With an expected effect size of d = 0.67 (based on previous studies), a sample size of 72 patients (36 per group) provides adequate power to detect clinically meaningful differences in eGFR change between groups.

11. QUALITY ASSURANCE

11.1 Quality Control Measures

- Regular monitoring of data quality and completeness
- Verification of laboratory results against source documents
- Duplicate measurements for key outcomes (e.g., urinary indoxyl sulfate analyzed in duplicate)
- Standardized procedures for all laboratory investigations
- Training of all study personnel on study protocols and procedures

11.2 Laboratory Quality Control

- Standard curves for ELISA assays constructed with R² >0.97
- Coefficient of variation for duplicate measurements <15%
- Regular calibration of laboratory equipment
- Use of quality control samples in each batch of assays

12. EXPECTED OUTCOMES AND SIGNIFICANCE

12.1 Expected Outcomes This study is expected to demonstrate that probiotic supplementation retard progression of disease in patients with moderate to severe CKD. The study will also provide evidence regarding the mechanisms underlying this effect, particularly whether it is mediated by reduction in uremic toxins such as indoxyl sulfate.

12.2 Significance

- Scientific Significance: This study will advance our understanding of the gut-kidney axis and the potential of microbiota modulation as a therapeutic target in CKD
- Clinical Significance: If positive, results will provide evidence for a novel, safe, and cost-effective disease-modifying intervention in moderate-stage CKD
- Public Health Significance: Given the high prevalence of CKD and limited therapeutic options, this intervention could have substantial public health impact

13. DISSEMINATION OF RESULTS AND PUBLICATION POLICY

13.1 Dissemination of Results

- Results will be disseminated through peer-reviewed scientific publications
- Findings will be presented at national and international scientific conferences
- Results will be communicated to study participants and their healthcare providers
- Findings will be made available to relevant health policy makers

13.2 Publication Policy

- The principal investigator (Dr. Samar Elshahat Saleh) will take the lead in manuscript preparation and submission
- All co-investigators will be acknowledged as co-authors based on their contributions
- Authorship will follow the International Committee of Medical Journal Editors (ICMJE) guidelines
- The study will be registered in ClinicalTrials.gov prior to enrollment of the first participant

14. ETHICS AND INFORMED CONSENT

14.1 Ethical Considerations

- The study will be conducted in accordance with the Declaration of Helsinki and Good Clinical Practice (GCP) guidelines
- The study protocol has been reviewed and approved by the Medical Research Ethics Committee of Mansoura Faculty of Medicine (Approval Code: MD.24.06.864, Date: July 14, 2024)
- All participants will provide written informed consent before enrollment
- Participant confidentiality will be maintained throughout the study
- Participants will be informed of their right to withdraw from the study at any time without providing a reason and without penalty

14.2 Informed Consent Process

- All eligible participants will be provided with a detailed informed consent form (ICF) in Arabic (local language)
- The ICF will explain the study objectives, procedures, risks, and benefits in clear, understandable language
- Participants will be given adequate time to read the ICF and ask questions
- Written informed consent will be obtained before any study procedures are performed
- A copy of the signed ICF will be provided to each participant

14.3 Risks and Benefits

- Risks: Probiotic supplementation is generally well-tolerated with minimal adverse effects. Potential risks include mild gastrointestinal symptoms (bloating, gas, diarrhea) in some individuals. These symptoms typically resolve spontaneously.
- Benefits: Participants may experience improvement in kidney function if the probiotic is effective. All participants will receive standardized medical care and close monitoring during the study.

15. DURATION OF THE PROJECT

Timeline:

- Month 1: Ethics approval, recruitment, and baseline assessment
- Months 2-7: Intervention period with follow-up visits at weeks 2, month 3, and month 6
- Month 7: Final data collection and study completion
- Months 8-9: Data analysis and manuscript preparation

16. PROBLEMS ANTICIPATED AND SOLUTIONS

| Anticipated Problem | Potential Solution |
| --- | --- |
| Low recruitment rate | Increase publicity within the clinic; extend recruitment period if necessary; consider recruiting from multiple centers |
| Poor treatment adherence | Regular telephone follow-up; provide adherence reminders; assess barriers to adherence at each visit |
| Loss to follow-up | Maintain updated contact information; provide transportation assistance if needed; send appointment reminders |
| Adverse events | Have emergency protocols in place; ensure participants know how to contact study team; have access to emergency medical care |
| Data quality issues | Implement regular data monitoring; provide training to study personnel; use standardized procedures |
| Laboratory equipment failure | Maintain service contracts; have backup equipment available; establish relationships with alternative laboratories |

17. PROJECT MANAGEMENT

| Team Member | Role | Responsibilities |
| --- | --- | --- |
| Dr. Samar Elshahat Saleh (PI) | Principal Investigator | Overall study design and conduct; participant recruitment; data collection; manuscript preparation |
| Dr. Nagy Sayed-Ahmed | Co-Investigator/Supervisor | Study supervision; critical review of protocol and manuscript; oversight of data quality |
| Dr. Sherouk Salah Eldin Elnagar | Co-Investigator | Data analysis; statistical interpretation; manuscript revision |
| Dr. Mohamed Ashraf Fouda | Co-Investigator | Data analysis; interpretation of results; manuscript revision |
| Independent Pharmacist | Allocation Concealment Manager | Maintenance of randomization codes; allocation concealment; code breaking in emergencies |
| Laboratory Staff | Laboratory Manager | Supervision of laboratory procedures; quality control; equipment maintenance |

PART 2: BUDGET AND RESOURCES

Note: Budget details are available upon request from the corresponding author.

Funding: This study is funded as part of an MD research program by the Urology and Nephrology Center, Mansoura University, Egypt. No external commercial or industry funding is received.

REFERENCES

- GBD Chronic Kidney Disease Collaboration. Global, regional, and national burden of chronic kidney disease, 1990–2017: a systematic analysis for the Global Burden of Disease Study 2017. Lancet. 2020;395(10225):709-733.
- Stenvinkel P, Carrero JJ, Axelsson J, Lindholm B, Heimbürger O, Massy Z. Emerging biomarkers for evaluating cardiovascular risk in the chronic kidney disease patient: how do new pieces fit into the uremic puzzle? Clin J Am Soc Nephrol. 2008;3(2):505-521.
- Vaziri ND. CKD impairs barrier function and alters microbial flora of the intestine: a major link to inflammation and uremic toxicity. Curr Opin Nephrol Hypertens. 2012;21(6):587-594.
- Jiang S, Xie S, Lv D, et al. Alteration of the gut microbiota in Chinese population with chronic kidney disease. Sci Rep. 2017;7:10676.
- Kieffer DA, Piccolo BD, Vaziri ND, et al. Resistant starch alters gut microbiome and metabolites and reduces atherosclerosis in apoE-KO mice. Mol Nutr Food Res. 2016;60(12):2518-2528.
- Vanholder R, Schepers E, Pletinck A, Nagler EV, Glorieux G. The uremic toxicity of indoxyl sulfate and p-cresyl sulfate: a systematic review. J Am Soc Nephrol. 2014;25(9):1897-1907.
- Lekawanvijit S. Cardiotoxicity of uremic toxins: a driver of cardiorenal syndrome. Toxins (Basel). 2018;7(5):1835-1848.
- Sirich TL, Plummer NS, Astor BC, Gipson DS, Hostetter TH, Meyer TW. Dietary potassium and sodium management in CKD: a practical approach and review of the literature. Int J Nephrol Renovasc Dis. 2016;9:37-49.
- Evenepoel P, Meijers BK, Bammens BR, Verbeke K. Uremic toxins originating from colonic microbial metabolism. Kidney Int Suppl. 2009 Dec;(114):S12-9. doi: 10.1038/ki.2009.402.
